# Structural analyses of NudT16–ADP-ribose complexes direct rational design of mutants with improved processing of poly(ADP-ribosyl)ated proteins

**DOI:** 10.1038/s41598-019-39491-w

**Published:** 2019-04-11

**Authors:** Puchong Thirawatananond, Robert Lyle McPherson, Jasmine Malhi, Sara Nathan, Michael J. Lambrecht, Matthew Brichacek, Paul J. Hergenrother, Anthony K. L. Leung, Sandra B. Gabelli

**Affiliations:** 1 Department of Biophysics and Biophysical Chemistry, Johns Hopkins University School of Medicine, Baltimore, MD 21205 USA; 2 Department of Biochemistry and Molecular Biology, Bloomberg School of Public Health, Johns Hopkins University, Baltimore, MD 21205 USA; 3 Department of Chemistry, University of Illinois, Urbana, IL 61801 USA; 4 Department of Oncology, Johns Hopkins University School of Medicine, Baltimore, MD 21287 USA; 5 Department of Molecular Biology and Genetics, Johns Hopkins University School of Medicine, Baltimore, MD 21205 USA; 6 Department of Medicine, Johns Hopkins University School of Medicine, Baltimore, MD 21205 USA

## Abstract

ADP-ribosylation is a post-translational modification that occurs on chemically diverse amino acids, including aspartate, glutamate, lysine, arginine, serine and cysteine on proteins and is mediated by ADP-ribosyltransferases, including a subset commonly known as poly(ADP-ribose) polymerases. ADP-ribose can be conjugated to proteins singly as a monomer or in polymeric chains as poly(ADP-ribose). While ADP-ribosylation can be reversed by ADP-ribosylhydrolases, this protein modification can also be processed to phosphoribosylation by enzymes possessing phosphodiesterase activity, such as snake venom phosphodiesterase, mammalian ectonucleotide pyrophosphatase/phosphodiesterase 1, *Escherichia coli* RppH, *Legionella pneumophila* Sde and *Homo sapiens* NudT16 (*Hs*NudT16). Our studies here sought to utilize X-ray crystallographic structures of *Hs*NudT16 in complex with monomeric and dimeric ADP-ribose in identifying the active site for binding and processing free and protein-conjugated ADP-ribose into phosphoribose forms. These structural data guide rational design of mutants that widen the active site to better accommodate protein-conjugated ADP-ribose. We identified that several *Hs*NudT16 mutants (Δ17, F36A, and F61S) have reduced activity for free ADP-ribose, similar processing ability against protein-conjugated mono(ADP-ribose), but improved catalytic efficiency for protein-conjugated poly(ADP-ribose). These *Hs*NudT16 variants may, therefore, provide a novel tool to investigate different forms of ADP-ribose.

## Introduction

ADP-ribosylation is a post-translational modification in which ADP-ribose (ADPr) is added onto glutamate, aspartate, cysteine, serine, lysine, and arginine residues of proteins using NAD^+^ as their substrate^1–5^. ADP-ribosylation is catalyzed by ADP-ribosyltransferases, including a family of 17 members in humans commonly known as poly(ADP-ribose) polymerases (PARPs)^6^. While five PARPs, including the founding member PARP1, add multiple ADPr units in a linear and/or branched fashion [poly(ADP-ribosyl)ation; PARylation], the majority of them (*e*.*g*., PARP10) modify with a single ADPr [mono(ADP-ribosyl)ation; MARylation]^5–8^. ADP-ribosylation is involved in diverse cellular functions, including DNA repair, transcription, chromosome segregation, cell cycle, cell metabolism, cell death and RNA metabolism^3,9–11^. In addition, this protein modification often acts as a scaffolding mechanism of other proteins^2^ in DNA damage repair^3,12–14^, mitotic spindle^15^ and RNA granule formation^11,16–18^. To elucidate relevant signaling mechanisms, it is critical to identify the sites of ADP-ribosylation and perform functional studies probing site-specific ADP-ribosylation^19–23^.

ADP-ribosylation can be reversed by ADP-ribosylhydrolases that cleave the glycosidic bonds between ADPr units and/or the bond between protein and ADPr^24,25^. Recent studies indicate that ADP-ribosylation can also be processed to phosphoribosylation *in vivo* by phosphodiesterases, such as SdeA in human pathogen *Legionella pneumophila*^24,26–29^, which cleave at the pyrophosphate bond within ADPr. A similar cleavage reaction was observed *in vitro* with other enzymes from diverse organisms, including a snake venom phosphodiesterase (SVP), mammalian ectonucleotide pyrophosphatase/phosphodiesterase 1 (ENPP1), *Escherichia coli* RppH (*Ec*RppH), and *Homo sapiens* NudT16 (*Hs*NudT16)^30–34^. Of note, from that list the Nudix enzymes demonstrated multiple substrate specificities *in vitro*. For example, *Hs*NudT16 hydrolyzes inosine triphosphate^35^ or diphosphate^36^ and is involved in a protective role in maintaining chromosome stability and cell growth by eliminating inosine-containing nucleotides in mammals^36^. In addition, although NudT16 was initially characterized as a nucleolar U8 snoRNA decapping enzyme^37,38^, it is involved in mRNA decapping and expresses across mouse tissues^39^.

Given the versatility in the recognition of substrates with diphosphate bonds^40,41^, we and others have used some of these Nudix enzymes *in vitro* for processing ADP-ribosylation to a unique mass tag for site identification by tandem mass spectrometry (MS/MS)^23,30–34^. Site identification has been a challenge for the field of ADP-ribosylation due to the heterogeneous nature of the number of ADPr units conjugated to protein. As a result, it is difficult to assign a unique mass signature associated with this protein modification. One possible solution is to treat MARylated and PARylated proteins/peptides with SVP, ENPP1 or NUDIX enzymes *Ec*RppH and *Hs*NudT16^23,30–34^, resulting in a 212.0086 Da phosphoribose tag at the otherwise modified residues. This phosphoribose tag allows for enrichment by phosphoproteomics approaches and site identification by mass spectrometry. However, it is unclear how *Hs*NudT16 processes different forms of ADP-ribosylation.

Here we report the crystal structures of *Hs*NudT16 in complex with ADPr and dimeric ADPr (di-ADPr). The structural data provides a rationale for the recognition and hydrolysis of protein-conjugated ADPr. By using this structural information, we designed mutants to better catalyze this hydrolysis reaction^42^ and evaluated them with hydrolysis assays for free ADPr, MARylated and PARylated substrates. Compared with the wild-type, *Hs*NudT16 mutants Δ17, F36A, and F61S have reduced hydrolysis activity towards free ADPr, comparable processing ability for MARylated proteins, and improved catalytic efficiency for PARylated proteins.

## Materials and Methods

### Cloning, subcloning, and mutagenesis

*Hs*NudT16 is encoded by NUDT16A (Uniprot Q96DEO) with an A22V mutation, which is present in the National Institute of Health’s Mammalian Gene Collection (accession no. BC031215)^43^. *Hs*NudT16 is subcloned in pNIC28-BSA4 (pNIC28-BSA4-*Hs*NudT16) such that the construct contains an N-terminal 6x-His-tag cleavable by Tobacco Etch Virus protease^43^. *Hs*NudT16 mutants H24W, F36A, F61S, F36A F61S and Δ17, which lacks the first 17 amino acids, were made by site-directed mutagenesis (GenScript).

### Expression and purification of NudT16

*Hs*NudT16 and its mutants were purified using an adapted protocol^43^. Briefly, a starter culture of LB supplemented with 50 µg/mL kanamycin and 34 µg/mL chloramphenicol was inoculated using a glycerol stock of CodonPlus RIPL *E*. *coli* cells (Agilent) that had been transformed with the pNIC28-BSA4-NudT16 plasmid and left to grow at 37 °C overnight. 10 mL of starter culture was used to inoculate each four 1 L cultures containing TB supplemented with 50 µg/mL kanamycin and 34 µg/mL chloramphenicol. After shaking at 37 °C and OD_600_ = 1 protein expression was induced with a concentration of IPTG of 1 mM. Cells grew at 18 °C shaking at 200 rpm overnight and were harvested by centrifugation at 5000 rpm. Cell pellets were resuspended in lysis buffer (50 mM NaH_2_PO_4_, 150 mM NaCl, 10 mM imidazole) were frozen at −80 °C.

Thawed cell resuspensions were lysed using a Microfluidizer primed to 80 psi. Cell lysates were clarified through centrifugation at 4 °C and 25412 × *g* for 45 minutes. Clarified supernatant were filtered using a 0.22 µm Stericup filter, and filtered supernatant was incubated with Ni-NTA beads at 4 °C for 2 hours. Incubated Ni-NTA beads were poured over a gravity column, washed with wash buffer (50 mM NaH_2_PO_4_, 150 mM NaCl, 20 mM imidazole), and were eluted with elution buffer (50 mM NaH_2_PO_4_, 150 mM NaCl, 50 mM imidazole). Fractions containing *Hs*NudT16 determined by SDS-PAGE were pooled, TEV protease was added in a ratio 1:100 TEV: protein to cleave the histidine tag and the mixture was dialyzed in 50 mM Tris-HCl, pH 8.0, 300 mM NaCl, 0.5 mM EDTA, and 1 mM DTT overnight at 4 °C.

The dialysis buffer was switched to 50 mM Tris-HCl, pH 8.0, 50 mM NaCl for 3 hours. The sample was clarified by centrifugation at 4000 × *g* and the resulting supernatant was syringe filter sterilized. The protein was loaded onto a Resource 15Q (GE Healthcare) anion exchange column equilibrated with Buffer A (50 mM Tris-HCl, pH 8.0, 50 mM NaCl) and eluted with a 0–100% gradient of Buffer B (50 mM Tris-HCl, pH 8.0, 50 mM NaCl). Fractions of interest determined by SDS-PAGE were pooled and dialyzed in 50 mM Tris-HCl, pH 8.0, 200 mM NaCl, 1 mM EDTA overnight at 4 °C.

Protein sample was syringe filter sterilized and concentrated to 5 mL using a Vivaspin20 10 kDa MWCO centrifugation concentrator. Concentrated protein sample was then loaded onto a HiLoad 26/600 Superdex 200 pg (GE Healthcare) size exclusion column equilibrated with 50 mM Tris-HCl, pH 8.0, 200 mM NaCl, 1 mM EDTA. Fractions of interest determined through SDS-PAGE were pooled, concentrated to 15–25 mg/mL and frozen at −80 °C for storage.

### Determination of Quaternary structure in solution of the *Hs*NudT16 variants

An additional step of size exclusion was performed using a Superdex 75 10/300 to determine whether the quaternary structure of *Hs*NudT16 is affected by H24W, F36A, F61S, F36A F61S or Δ17 mutation. 100 µL of each purified *Hs*NudT16 mutant was loaded onto the column at a rate of 0.3 mL/min and was eluted over one column volume. Size exclusion standards from Bio-Rad (catalog number: 151–1901) were run using the same method.

### Crystallization of *Hs*NudT16

Crystallization conditions for *Hs*NudT16 and its mutants were found using the QIAGEN JCSG I and Molecular Dimensions Shotgun commercial screens set with a TTP mosquito robot (TTP Labtech). Crystals of *Hs*NudT16 were grown via hanging drop vapor diffusion at 293 K. Drops were made by mixing 1 µL of *Hs*NudT16 (26 mg/mL) containing 2 mM ADPr with 1 µL of 0.1 M Tris-HCl pH 8.5, 0.2 M sodium acetate trihydrate, and 25% PEG 4000 by vapor diffusion. Prior to data collection, crystals were soaked with 4 mM ADPr or 1 mM di-ADPr for 5 hours for the *Hs*NudT16-ADPr and *Hs*NudT16-di-ADPr structures, respectively^44^. di-ADPr was chemically synthesized and purified as described before^44^.

Each of the purified mutants concentrated to 15 mg/mL were mixed with 2 mM ADPr prior to setting up the trays. *Hs*NudT16 mutants F36A and H24W were crystallized in 0.1 M CHES, pH 9.5 and 20% PEG 8000. *Hs*NudT16 F61S in complex with ADPr was crystallized in multiple conditions, but the condition for which data was collected was 0.1 M HEPES, pH 7.5 and 20% PEG 8000. Crystallization conditions were further optimized by hanging drop vapor diffusion by fixing the pH and varying PEG 8000 between 15–25% in 24-well trays.

### Data collection, structure determination and refinement

Data for native *Hs*NudT16 protein crystals in complex with ADPr and with di-ADPr and all *Hs*NudT16 mutants were collected on a FR-e Super-Bright (Rigaku Americas Corporation, The Woodlands, TX) copper rotating anode x-ray generator as the source with a DECTRIS Pilatus 3R 200K-A detector at 100 K (−173 °C). Data were processed with HKL3000. The structure of the *Hs*NudT16 in complex with ADPr (*Hs*NudT16-ADPr) was determined by molecular replacement using PDB ID 3COU as a template^43^. The *Hs*NudT16 in complex with di-ADPr (*Hs*NudT16-di-ADPr), *Hs*NudT16 61A, *Hs*NudT16 H24W, *Hs*NudT16 F36A structures were determined by Fourier synthesis using our initial structure (PDB ID 6B09, 5WJI, 5W6Z, 5VY2, respectively). Each of the models was rebuilt and refined using alternate cycles of Coot and restrained refinement with Refmac5 in the CCP4 suite^45,46^. During rebuilding and refinement of the model *Hs*NudT16-ADPr, the identity of the metal atoms (sodium vs magnesium) was determined based on the distance and number of ligands using databases^47–51^. Sodium atoms were the ones that refined best^50,52^. Water molecules were discarded in such position since they should have less number of ligands and the distance water-molecule ligand would be longer. In the deposited structure, PDB ID 5W6X, the metal binding sites are occupied by a sodium atom with a 6-ligand coordination and the shortest distance of 2.25–2.35A. Final models were validated using Coot^45^, Molprobity^53^ (Table 1). Structure figures were prepared with PyMOL^54,55^.

**Table 1:** Data collection and refinement statistics for *Hs*NudT16 and its mutants.

	ADPr (PDB ID 5W6X)	H24W (PDB ID 5W6Z)	F36A (PDB ID 5VY2)	F61S (PDB ID 5WJI)	di-ADPr (PDB ID 6B09)
**Data collection**					
Space group^ℑ^	C2	P2_1_	P2_1_	C2	C2
**Cell dimensions**					
*a*, *b*, *c* (Å)	113.7, 46.3, 74.2	50.1, 119.4, 65.8	57.2, 49.6, 64.3	113.8, 47.0, 72.3	113.3, 47.0, 75.7
α, β, γ (°)	90.0, 107.7, 90.0	90.0, 90.7, 90.0	90.0, 114.6, 90.0	90.0, 110.4, 90.0	90.0, 108.7, 90.0
Resolution (Å)	70.74-2.10 (2.18-2.10)	65.77-2.61 (2.64-2.61)	58.45-2.30 (2.34-2.30)	60.00-2.30 (2.34-2.30)	71.7-3.14 (3.21-3.14)
Wavelength (Å)	1.542	1.000	1.542	1.542	1,542
R_symm_ (%)	6.0 (20.8)	12.0 (26.1)	7.0 (13.5)	6.7 (34.2)	13.2 (52.9)
I/Sigma	26.6 (5.3)	83.7 (42.9)	31.1 (10.0)	23.5 (2.2)	8.04 (1.95)
Completeness (%)	88.0 (46.5)	90.1 (52.4)	92.0 (74.3)	90.8 (47.9)	91.4(95.5)
Unique Reflections	21,917	23,461	14,915	16,472	6,140
Total Reflections	61,145	64,806	34,703	41,814	13,751
Redundancy	3.2 (2.0)	3.1 (2.4)	2.5 (1.7)	2.8 (1.4)	2.2 (2.1)
**Refinement**					
R_work_/R_free_ (%)	0.18/0.24	0.24/0.31	0.19/0.25	0.20/0.27	0.25/0.32
**No**. **atoms**					
Protein	2,784	5,640	2,787	2,740	2,749
Ligand	76	1	1	11	4 Mg, 186
H_2_O	266	67	176	83	8
**B-factors**					
Protein	30.1	45.5	30.1	49.1	55.1
Water	35.0	30.0	44.2	47.5	23.7
Ligand	47.8	31.4	29.8	82.5	78.5
**RMSD**					
Bond lengths (Å)	0.015	0.015	0.011	0.013	0.012
Bond angles (°)	0.002	0.002	0.002	0.002	0.007

^ℑ^Analysis of crystal packing does not reveal a symmetry-related molecule in the binding site and the packing does not appear to prevent diffusion of substrates in the crystals.

### Michaelis-Menten Kinetics

Hydrolysis of free ADPr by *Hs*NudT16 and its variants were measured with Malachite Green phosphatase detection kit (R&D Systems catalog DY996). Calf intestinal phosphatase (CIP; NEB; catalog: M0290L), coupled with the Nudix reaction hydrolyzes the products of the Nudix reaction (phosphoribose and AMP) into 2P_i_, adenosine, and ribose^56^. A phosphate standard curve of 0–100 μM was made through serial dilutions of 1 M KH_2_PO_4_. Michaelis-Menten analysis was performed for *Hs*NudT16 and mutants H24W, F36A, F61S, F36A F61S and Δ17. Each reaction mix consisted of 1 mL of 50 mM Tris-HCl, pH 8.0, 150 mM NaCl, 2 mM MgCl_2_, 0–5 mM ADPr, 10 U of CIP, and 100 nM *Hs*NudT16 enzyme (or one of the mutants). Substrate concentrations ranged from 0–4.5 mM. After the initiation of the reaction by the addition of *Hs*NudT16, 150 μL time point samples were taken every 4 minutes for 20 minutes and quenched with 5 μL of 300 mM EDTA (final concentration ~10 mM). 50 μL aliquots of each time points were pipetted into a 96-well clear Grenier plate into individual wells as triplicates to which 10 μL of ammonium molybdate in 3 M sulfuric acid was added and incubated at room temperature for 10 minutes. Afterwards, 10 μL of malachite green oxalate and polyvinyl alcohol was added and incubated at room temperature for 5 minutes to allow color development and the A620 was read with a Tecan microplate reader.

### PARP10^CD^ Demodification Assay by *Hs*NudT16 and its mutants

PARP10^CD^, the catalytic domain of PARP10 (amino acids 818–1025), was purified as previously described^57^. For each reaction, 1 μg PARP10^CD^ was automodified (auto-MARylated) with 0.5 μCi ^32^P-NAD^+^ for 30 min at 30 °C in automodification buffer (20 mM Tris-HCl pH 7.5, 50 mM NaCl, 5 mM MgCl_2_, 10 mM β-mercaptoethanol). Excess ^32^P NAD^+^ was removed by desalting by gravity flow in a Micro Bio-spin Column (Bio-Rad) into *Hs*NudT16 reaction buffer (20 mM Tris-HCl pH 7.5, 50 mM NaCl, 5 mM MgCl_2_, 10 mM β-mercaptoethanol). ^32^P-labeled MARylated PARP10^CD^ was incubated with 1 μg of *Hs*NudT16 or buffer alone in a 12 μL reaction at 37 °C for indicated time points. Reactions were stopped with SDS/PAGE Laemmli Buffer 2X, and samples were subjected to SDS/PAGE on a 15% (wt/vol) Tris-Glycine gel. Total protein levels were analyzed with SimplyBlue Safe Stain (Invitrogen), and ^32^P signal was visualized by autoradiography. For quantification, signal intensity of the radioactive band corresponding to MARylated PARP10^CD^ was quantified in ImageJ image analysis software and compared to the intensity at the 0 min time point.

### PARP1 Demodification Assay by *Hs*NudT16 and its mutants

Full-length *Hs*PARP1 was purified as previously described^30^. For each reaction, 1 μg PARP1 was auto-PARylated with 0.5 μCi ^32^P NAD^+^ for 30 min at 37 °C in automodification buffer supplemented with 100 μM NAD^+^ and 1 μM annealed DNA. For time-course experiments, ^32^P-labeled PARylated PARP1 was incubated with 1 μg of *Hs*NudT16 proteins at 37 °C in a 10 μL reaction for indicated time points. For dose-dependent experiments, ^32^P-labeled PARylated PARP1 was incubated with indicated amount of *Hs*NudT16 proteins in a 15 μL reaction at 37 °C for 10 min. For quantification, signal intensity of the radioactive smear for PARylated PARP1, defined as the area between the well and unmodified PARP1 for each lane, was quantified in ImageJ image analysis software and compared to the intensity at either the 0 min time point or no protein control.

### Accession Codes

Atomic coordinates and structure factors for the *Hs*NudT16-ADPr (PDB ID: 5W6X), *Hs*NudT16-di-ADPr (PDB ID: 6B09), *Hs*NudT16 H24W (PDB ID: 5W6Z), *Hs*NudT16 F36A (PDB ID: 5VY2), and *Hs*NudT16 F61S (PDB: ID 5WJI) were deposited in the Protein Data Bank.

## Results

### Structural determinants of ADP-ribose recognition by *Hs*NudT16 are buried in the core of the enzyme

**Figure 1:**
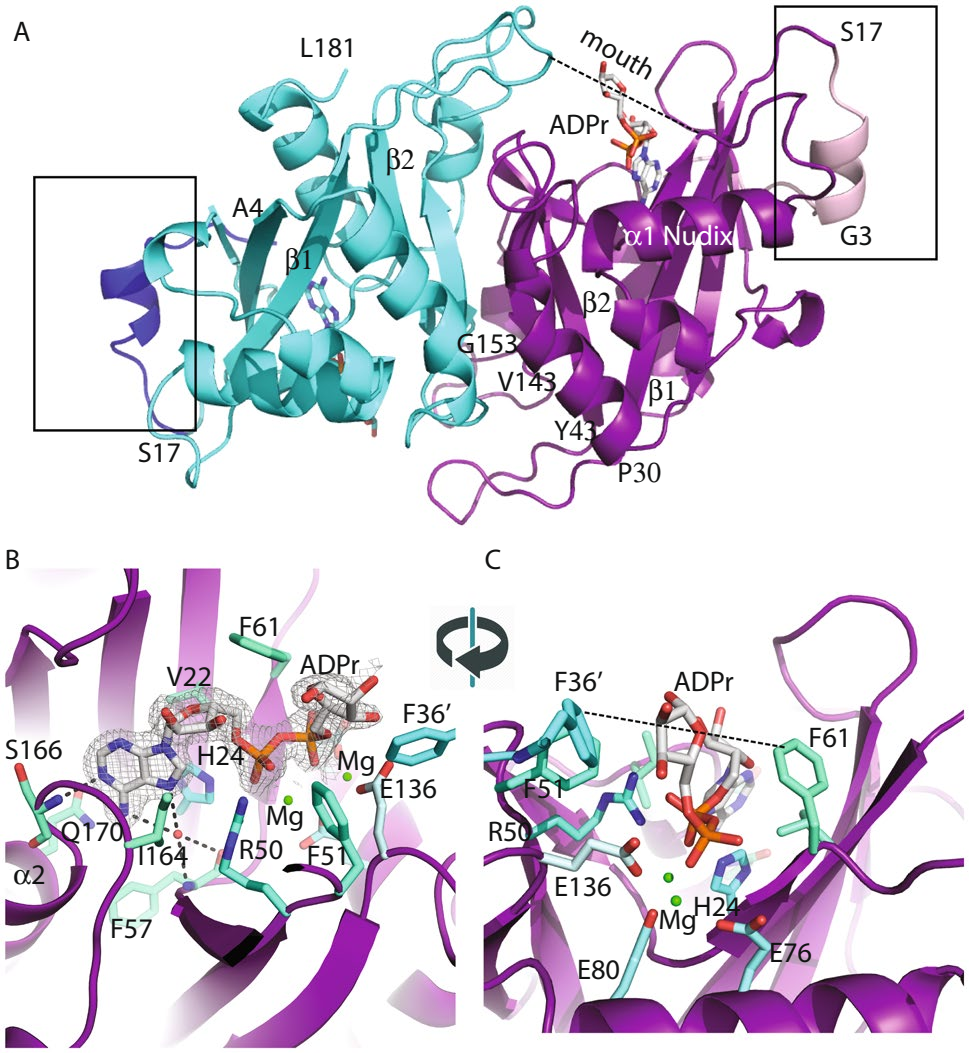
Identification of the ADP-ribose binding site in *Hs*NudT16. (**A**) Structure of ADPr bound to the active site of *Hs*NudT16. The dimer displays a 2-fold axis—one monomer is colored in cyan with amino acids 4–17 in dark blue (box); the other monomer is in purple with amino acids 3–17 in light pink (box). ADPr is shown as white sticks. A dashed line from Phe36′ to Phe 61 of the opposite monomer delineates the “mouth” of the active site. (**B**) 2Fo-Fc Electron density map of the bound ADPr contoured at 1σ (gray). The secondary structure of *Hs*NudT16 is shown in purple and residues that delimit the binding site are shown as sticks in aquamarine. F36′ from the other monomer is shown in cyan. (**C**) about 90° view from (**B**).

**Figure 2:**
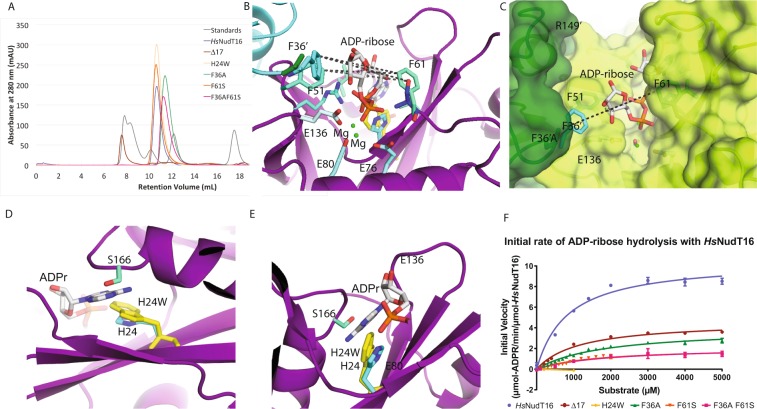
Biophysical characterization of *Hs*NudT16 mutants and their hydrolytic activity towards free ADPr. (**A**) Size exclusion chromatograms of the *Hs*NudT16 (shown in blue), Δ17 (brown), H24W (golden), F36A (green), F61S (orange) and F36A F61S (pink) mutants. Elution volume of H24W and F61S indicate the preservation of their quaternary state as dimers. Molecular weight standards (Bio-Rad catalog number 151–1901) are shown in gray (peaks from left to right represent 670 kDa, 158 kDa, 44 kDa, 17 kDa and 1.35 kDa, respectively). (**B**) Structure of *Hs*NudT16 with one monomer shown in cyan and the other in purple. ADPr is shown in white sticks in the active site. Dashed line shows the distance from F61 of one monomer to F36′ of the other at 9.7 Å at the CE atom; the distance of the mutant F61S OG atom to the F36 to be 10.7 Å; (**C**) Structure of F36A mutant with its monomers shown in dark green and green (PDB ID: 5VY2), overlaid with one monomer of *Hs*NudT16 shown in cyan and its respective ADPr with white carbon atoms (PDB ID: 5W6X) aligned on the dark green chain (only the side chain of F36′ is observed). Dashed line shows the widening of the distance to 12.6 Å in the F36A mutant (below the green surface) to the F61. (**D**) Structure of H24W mutant (PDB ID: 5W6Z) shown in purple zoomed in to the binding pocket with the ADPr from the *Hs*NudT16 structure (PDB ID: 5W6X) in white sticks modeled in the binding site; the tryptophan residue in yellow with the modeled ADPr in white demonstrates the occlusion of the substrate by the Tryptophan side chain in the binding site. (**E**) Same as (**D**) but rotated nearly 180°. (**F**) Comparison of the ADPr hydrolase activity of *Hs*NudT16 and its mutants. Michaelis-Menten plots are shown from 0 to 5 mM ADPr. Activity (V_max_) is expressed in μmol-ADPr/min. Error bars represent SD, n = 4.

The crystals of *Hs*NudT16 in complex with ADPr diffract to a resolution of 2.1 Å, contain a non-crystallographic dimer in the asymmetric unit and display clear electron density for the ADPr in each monomer binding site (Fig. 1, Supplementary Fig. S1). The dimer interface buries about 1300 Å^2^ with the loops connecting strands β1 with β2 (aa 30–43) and strand β7 with helix α2 (aa 143–153) reaching to the opposite monomer (Fig. 1A, Supplementary Fig. S2). The first 17 amino acids, an addition to the minimal Nudix fold, flank the fold at each side (Fig. 1A, boxed area). Each *Hs*NudT16 monomer has a molecule of ADPr bound burying about 470 Å^2^ out of the total 660 Å^2^ of accessible surface as calculated with PISA^58^. The *Hs*NudT16-ADPr complex structure displays the purine ring of the ADPr buried deep in the binding site stacked between His24 and Ile164 (Fig. 1B, Supplementary Fig. S1). The amine (NH2) of the purine ring is at hydrogen bond distance of Gln170 and Ser166, both residues of the α2 helix. The pocket where the adenosine binds is positively charged in sharp contrast with the negatively charged pocket formed by the glutamate residues of the Nudix sequence, ${G}_{1}^{N}[5X]{E}_{7}^{N}[7X]{R}_{15}^{N}{E}_{16}^{N}{XX}{E}_{19}^{N}{E}_{20}^{N}X{G}_{22}^{N}U$, Glu76, Glu80 and Glu136 (${E}_{16}^{N}$, ${E}_{19}^{N}$ and ${G}_{22}^{N}\,+\,50$) that bind the metal (Supplementary Fig. S1A–C). The oxygen in the α-phosphate of the ADPr is bridged to the glutamate residues of the Nudix sequence by magnesium atoms (Fig. 1B,C) and is at hydrogen bonding distance of the guanidinium of Arg50 on the opposite side. Therefore, the orientation of the diphosphate is in alignment to be hydrolyzed by a water molecule activated by the Mg^2+^ bound to Glu76, Glu80 and Glu136 (Supplementary Fig. S1B). Interestingly, the oxygen of the α-phosphate is also at hydrogen bonding distance of the His24. Using information from other enzymes in the Nudix superfamily, Glu 76, ${{\rm{E}}}_{16}^{{\rm{N}}}$ is the likely catalytic base, as in *Ec*MutT^59^, where ${{\rm{E}}}_{16}^{{\rm{N}}}$ and other glutamate residues orient two of the divalent cations. The non-adenosine ribose is solvent exposed, loosely held in the “mouth” of the protein formed by Phe61 on one side and Phe36 of the other monomer, lacking specific hydrogen bonds. This mouth is about ~ 9 Å in width from Phe36′ (of the opposite monomer) to Phe61 (dashed lines in Figs 1 and 2). The widening of this mouth could potentially allow protein molecules that are conjugated to ADPr to come closer to the active site. The *Hs*NudT16-ADPr complex arranges the substrate similarly to the *Hs*NudT16-IMP (PDB ID 2XSQ) as judged by the position of the scissile phosphodiester bond relative to Arg50 and the magnesium atoms^43^. At the same time, the adenosine base is recognized fully by residues of helix α2 while the inosine N1 and O6 atoms make hydrogen-bonds to Gln170 from helix α2 and the main chain of Phe57 (through a water molecule), most likely to avoid an unfavorable interaction^43^. The *Hs*NudT16-ADPr complex aligns structurally to the dimer of the apo *Hs*NudT16 (PDB ID 3MGM) with an rmsd of 0.6 over 240-carbon atoms over the 2 monomers (Supplementary Fig. S2C)^60^. The slight differences between the structures can be attributed to a ligand-induced conformational change on one side of the mouth of the enzyme. The changes are in the conformation and displacement of the loops that define the ‘mouth’: on amino acids 60–69, (~5 Å) and on the amino acids 100–110, (~4 Å, Supplementary Fig. S2B,C). Specifically, in the absence of ADPr, Phe61 moves in towards the active site where ADPr binds. Taken together, the structure of the *Hs*NudT16-ADPr complex suggests that the hydrolysis of free and protein-conjugated ADPr is dependent on the Nudix signature for catalysis and that the recognition of a particular substrate is mediated through distal structural elements, as has been studied in other Nudix families^40,61,62^. *Hs*NudT16 positions ADPr in reverse orientation compared to other members of the Nudix family of ADPrases (*Hs*NudT5, *Ec*ADPrase, *Mt*ADPrase)^40,63,64^ (Supplementary Fig. S1D–G). *Hs*NudT16 buries the adenine of ADPr close to the core of the enzyme and leaves the non-adenosine ribose exposed to the solvent (Supplementary Fig. S1D) while the Nudix ADPRases bind the ADPr by burying the non-adenosine ribose and keeping the adenine exposed (Supplementary Fig. S1E)^40,63,64^. Comparing how the two enzymes classes bind the substrate, the diphosphate of ADPr bound to these Nudix enzymes remains relatively the same position (Supplementary Fig. S1F). Notably, the arrangement of substrate in the binding site of *Hs*NudT16 allows the non-adenosine ribose to be exposed and can be conjugated to a protein^31^.

### Rational design of *Hs*NudT16 mutants to enhance the hydrolysis of protein-conjugated ADP-ribose

Based on the *Hs*NudT16-ADPr complex structure, we designed mutants tailored for the demodification of protein-conjugated ADPr. *Hs*NudT16 recognition of MARylated proteins could be particularly challenging since having just one ADPr would position a putative large molecule (protein) too close to the binding site and generate steric hindrance. PARylated proteins, on the other hand, present longer chains and therefore the protein is further away from the catalytic site. F36A and F61S mutants were designed to enlarge the mouth of the enzyme with the aim of easing the recognition and processing of the ADPr conjugated to proteins (Fig. 1). Modeling a Ser side chain at F61 position suggested that the mutated hydroxymethyl group could potentially come into hydrogen bonding distance to the non-adenosine ribose of the substrate, which may contribute to its orientation for catalysis. In addition, we made a double mutant F36A F61S in an attempt to combine these effects synergistically (Fig. 1C, Supplementary Fig. 2C). Observing that His24 came within close proximity to the adenine base, we also designed H24W mutant to improve base stacking (Fig. 1B,C). Lastly, we deleted the first 17 amino acids of the protein (Δ17) to explore the possibility that the N-terminal residues regulate hydrolysis by blocking the active site as has been suggested^65^ (Fig. 1A).

### Structural Basis of the *Hs*NudT16 mutants activity towards ADPr

The mutants of *Hs*NudT16 were successfully purified to 98% or better as determined by SDS-PAGE (Supplementary Fig. S3A). H24W and F61S mutants behave as dimers in solution as shown by the size-exclusion chromatography profile similar to *Hs*NudT16 (Fig. 2A). Interestingly, mutation of residue F36A results in a displacement of retention volume from ~10.5 ml (*Hs*NudT16, *Hs*NudT16 H24W and *Hs*NudT16 F61S), which correspond to a molecular weight of 40 kDa, to a retention volume of ~11.2 mL (*Hs*NudT16 F36A and F36A F61S), which correspond to a molecular weight of 27 kDa. This change in retention volume suggests that the lack of phenylalanine at position 36 weakens the dimer, shifting the population to a monomer. In accordance to this shift, analysis of buried surface upon dimerization shows a reduction from about 1320 Å^2^ in *Hs*NudT16 to about 1210 Å^2^ in *Hs*NudT16 F36A, where residues 33–36, 50–56 and 120–162 are defined as part of the dimeric interface (Supplementary Figs S2A,B, S3). The Δ17 mutant was unstable in low salt conditions and appeared as a larger aggregate at high concentration. While the unstable Δ17 mutant could not be concentrated above 1.7 mg/mL, the other *Hs*NudT16 mutants—H24W, F36A, F61S, and F36A F61S—were purified and concentrated to >15 mg/mL. Conditions of crystallization were found for the H24W, F36A, and F61S mutants and data were collected to 2.6, 2.3, and 2.5 Å, respectively (Table 1). Despite multiple co-crystallization attempts and soaking efforts, ADPr was not observed in these structures.

The crystal structures of mutants F36A and F61S show that the replacement of the phenyl side chain of these residues with a methyl or a hydroxymethyl group, respectively, opens up the mouth of the enzyme (Fig. 2, Supplementary Fig. S2B,C, S3). The structural overlap of the *Hs*NudT16-ADPr complex structure with the F36A structure shows that the binding pocket opens up close to the ribose by about 3 Å (Fig. 2B,C). The ~9 Å width of the cavity at the Phe36′ to Phe61 increases to 12.6 Å in the F36A mutant (Fig. 2C). The structural overlap of the *H*sNudT16-ADPr complex structure with the *Hs*NudT16 F61S structure shows that the serine side chain opens the mouth only by about 1 Å (Supplementary Fig. S3B,C). Specifically, the distance from Phe36 CE2 to the Phe61 CD2 is about 9.8 Å and to the hydroxyl of the F61S mutant opens up only to 10.8 Å (Fig. 2B, Supplementary Fig. S3B,C). Contrary to our hypothesis, the structure of the *Hs*NudT16 H24W shows that the indole ring of the tryptophan, instead of improving stacking, protrudes into the adenosine binding site, as demonstrated by structurally aligning with the *Hs*NudT16-ADPr model (Fig. 2D,E).

### Free ADP-ribose hydrolysis is reduced by Δ17, F36A, F61S, and F36A F61S mutations while H24W is inactive

To assess the effects of the mutations, the kinetic constants of the purified *Hs*NudT16 Δ17, H24W, F36A, F61S and F36A F61S mutants were determined by fitting with the Michaelis-Menten equation based on the activities of these enzymes towards various concentrations of ADPr. The steady-state hydrolysis assays were assessed colorimetrically by Malachite-green based assays to quantify free phosphate (Fig. 2F, Table 2). The V_max_ of *Hs*NudT16 towards ADPr was reduced at least 2-fold as a result of the selected mutations. The *K*_m_ for ADPr was also increased, suggesting that the mutants have a reduced binding affinity for ADPr. H24W mutant had no significant activity towards free ADPr; consequently, the *K*_m_ was not measured. The lack of enzymatic activity could be explained by the fact that the indole group of the tryptophan occupies the site of the adenosine and sterically hinders ADPr binding. This is compounded with the loss of interaction between the imidazole ring and the oxygen of the β-phosphate. The reduced activity of the Δ17 mutant is harder to rationalize. In the structures that we have determined, residues 1–17 (except Ala12) are solvent accessible and in the same conformation. The lack of change in the conformation and the diminished activity suggests that the deletion exposes a hydrophobic patch on the surface (Supplementary Fig. S4). Widening the mouth of *Hs*NudT16 at the level of the non-adenosine ribose seems to be deleterious for ADPr hydrolysis activity since the F36A mutant is less active. On the other hand, though the F61S mutant does not change significantly the width of the protein mouth, the activity towards free ADPr is decreased, suggesting that Van der Waals interactions between the phenylalanine side chain and the ribose reduce the possible conformational changes of the bound ligand and thus facilitate ADPr binding. No synergism is observed in the double mutant F36A F61S, suggesting that Phe36 and Phe61 play an insignificant role in the active site apart from steric hindrance.

**Table 2:** Comparative kinetic analysis of the ADP-ribose hydrolysis activity of *Hs*NudT16 and the designed mutants.

Enzyme	*K*_m_ (μM ADPr)	*k*_cat_ (s^−1^)
*Hs*NudT16	837 ± 174	0.176 ± 0.011
*Hs*NudT16 Δ17	1189 ± 301	0.083 ± 0.009
*Hs*NudT16 F36A	1818 ± 198	0.066 ± 0.004
*Hs*NudT16 F61S	1622 ± 330	0.035 ± 0.003
*Hs*NudT16 H24W	n/a	n/a
*Hs*NudT16 F36A-F61S	1880 ± 739	0.036 ± 0.006

The Michaelis-Menten constant ‘*K*_m_’ and maximum velocity ‘V_max_’ for the production of PO_4_ were estimated from non-linear regression by fitting the reaction velocities to Michaelis-Menten equation. *K*_m_ and V_max_ values are shown as mean ± SEM using the program GraphPad Prism Version 5.01. Data are representative of three independent experiments each performed in duplicate.

### *Hs*NudT16 Δ17, F36A, F61S, and F36A F61S mutants have a comparable demodification activity of MARylated PARP10^CD^ to that of *Hs*NudT16, although the H24W mutant is inactive

**Figure 3:**
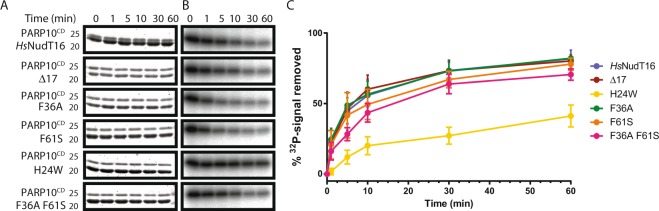
Comparison between *Hs*NudT16 and its mutants in their ability to process MARylated PARP10^CD^. (**A**) Coomassie blue-stained SDS gel of ^32^P-MARylated PARP10^CD^ (top band) demodification reaction by *Hs*NudT16 and mutants Δ17, F36A, F61S, F36A F61S and H24W displaying comparable amount of protein PARP10^CD^ at every time point. The positions of the molecular weight standards corresponding to 25 and 20 kDa are shown on the left. (**B**) ^32^P autoradiograph of the reactions in (**A**) displaying a decrease in radioactivity as time elapse. (**C**) Quantification of removal of ^32^P-radioactive signal from PARP10, n = 3. Please note that the apparent activity of H24W observed is due to the background hydrolysis of the MARylated substrates as shown in the buffer control (Supplementary Fig. S5).

To determine the hydrolysis efficacy of *Hs*NudT16 and its mutants towards MARylated proteins, demodification assays were performed on the catalytic domain of PARP10 (PARP10^CD^) which was pre-incubated with ^32^P-NAD^+^ to generate ^32^P-MARylated PARP10^CD^ as a substrate (Fig. 3). ^32^P-MARylated PARP10^CD^ was incubated with *Hs*NudT16 and its mutants for 0, 1, 5, 10, 30, and 60 minutes, and the products were run on an SDS-PAGE gel and visualized with Coomassie blue (Fig. 3A) and autoradiography (Fig. 3B). The demodification assay by *Hs*NudT16 hydrolyses the diphosphate bond within the ADP-ribose of ^32^P-MARylated PARP10^CD^, resulting in phosphoribosylated PARP10^CD^ and releasing ^32^P-AMP. As a result, the *Hs*NudT16-catalyzed hydrolysis did not significantly alter the gel mobility of PARP10^CD^ (top band; Fig. 3A) because of the relatively small change in molecular weight from single ADPr (558 Da) to phosphoribose (212 Da). However, time-dependent reduction of autoradiograph signal intensity was observed as P^32^-labeled ADPr modification on PARP10^CD^ is hydrolyzed by *Hs*NudT16 over the time course of 60 minutes (Fig. 3B). When compared with the *Hs*NudT16, no appreciable differences in signal reduction were observed with either Δ17, F36A, F61S or F36A F61S (Fig. 3B,C). In contrast, H24W mutant had little activity towards MARylated PARP10^CD^, in which the activity is comparable to the buffer control (Supplementary Fig. S5).

### Demodification of PARylated PARP1 is more efficient with the Δ17, F36A, F61S, and F36A F61S mutations but is ablated by the H24W mutation

**Figure 4:**
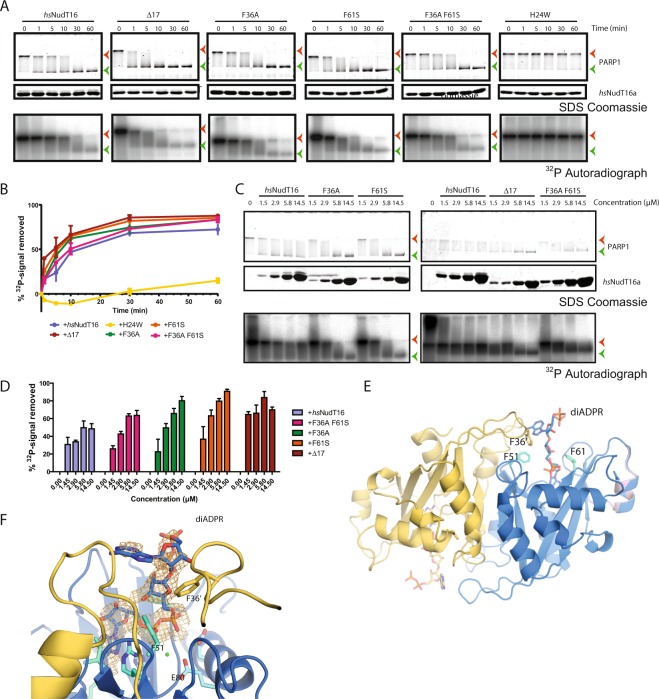
*Hs*NudT16 mutants Δ17, F36A, and F61S process PARylated PARP1 faster and at a lower concentration than the *Hs*NudT16. (**A**) Time course of the demodification reaction of ^32^P-PARylated PARP1 by *Hs*NudT16 and its mutants Δ17, F36A, F61S, F36A F61S and H24W as revealed by Coomassie blue-stained SDS-PAGE gels. Orange arrowheads indicate the interface between stacking and resolving gels where PARylated PARP1 was. Green arrowheads indicate unmodified PARP1. ^32^P autoradiographs of the demodification reaction were shown in lower panels. (**B**) Quantification of the removal of ^32^P-radioactive signal from the PARP1 demodification assay over time, n = 3. (**C**) The demodification of PARylated PARP1 by *Hs*NudT16 and its mutants Δ17, F36A, F61S and F36A F61S as revealed by Coomassie blue-stained SDS-PAGE gels and ^32^P autoradiograph as in (**A**). (**D**) Quantification of the removal of ^32^P-radioactive signal from the dose-dependent PARP1 demodification assays, n = 3. Though the volumes of the demodification reaction in the two panels are different (10 μL in panel B and 15 μL in panel D), the final amount of PARP1 loaded in each lane is the same across the two panels for comparison. (**E**) Structure of *Hs*NudT16 from crystals soaked with dimeric ADPr. The width of the “mouth” of the binding site between F36′ and F61 is marked with a dashed line. (**F**) Zoom-in view of the substrate in the binding site with an omit electron density map contoured at 1.5 σ (orange).

To determine the hydrolysis efficacy of *Hs*NudT16 and its mutants towards PARylated proteins, demodification assays were performed on the full length PARP1 which was pre-incubated with ^32^P-NAD^+^ to generate ^32^P-PARylated PARP1 as a substrate (Fig. 4). ^32^P-PARylated PARP1 was incubated with *Hs*NudT16 and its mutants for 0, 1, 5, 10, 30, and 60 minutes, and the products were run on an SDS-PAGE gel and were visualized with Coomassie blue and autoradiography (Fig. 4A). In this demodification assay, *Hs*NudT16 hydrolyses the diphosphate bonds in the P^32^-PARylated protein, thereby removing the ADP-ribose units in the form of ^32^P-containing *iso-*ADP-ribose and the terminal ^32^P-containing AMP. The full length unmodified PARP1 is a 113 kDa protein (green arrowheads); when PARylated, PARP1 appear as a smear due to the addition of heterogenous number of ADPr units and hence an increase in molecular weight, where most of the highly PARylated PARP1 was unresolved at the interface between stacking gel and resolving gel (orange arrowheads). Incubation of PARylated PARP1 with *Hs*NudT16 leads to a time-dependent downward shift in the Coomassie blue staining corresponding to the removal of ADPr units and an increase in gel mobility of PARP1 (Fig. 4A). Consistently, autoradiographs showed a signal reduction of the PARylated PARP1 (Fig. 4A). In time-dependent assays, the Δ17 mutant was able to demodify the PARylated PARP1 more efficiently than the *Hs*NudT16 as demonstrated by the faster reduction in radioactive signals, which is consistent with an earlier downward shift from PARylated PARP1 (lower gel mobility) to unmodified PARP1 (higher mobility) in the Coomassie blue-stained protein gel (Fig. 4A,B). By contrast, the F36A, F61S, and F36A F61S mutants had only a slight improvement and the H24W mutant is inactive towards PARylated PARP1 (Fig. 4A,B).

To further probe the differences in activities amongst various mutants, we performed the demodification assay with an increasing concentration of enzymes incubated for 10 minutes when *Hs*NudT16 removes 50% of radioactive signals from ^32^P-PARylated PARP1 (Fig. 4C,D). In this dose-dependent assay, Δ17, F36A, and F61S mutants outperformed *Hs*NudT16. Though we observed that the *Hs*NudT16 reduced the radioactive signals, most of the signals still remained at the interface between stacking and resolving gels at this timepoint (orange arrowheads in Fig. 4C). On the other hand, Δ17, F36A, and F61S mutants reduced the radioactive signals and PARP1 was migrated into the resolving gel (Fig. 4C). These data suggest that these mutants are more efficient in degrading the PAR polymer and thus faster in reducing the molecular weight associated with PARP1 (Fig. 4C,D). In contrast, the F36A F61S mutant showed relatively little improvement over *Hs*NudT16 (Fig. 4C,D), suggesting that the re-engineered active site of this double mutant does not display additive or synergistic effects.

### 2′-OH terminus of dimeric ADPr binds towards the edge of the Nudix fold of *Hs*NudT16

To better understand the binding of poly(ADP-ribose) (PAR) and the potential mechanism of demodification of PARylation, we determined the structure of *Hs*NudT16 with dimeric ADPr (di-ADPr) (PDB ID: 6B09; Fig. 4E,F). The orientation of the first ADPr at the 2′-OH terminal end of the dimer matches that of the *Hs*NudT16-ADPr structure (PDB ID: 5W6X) with the adenosine base buried in the binding pocket and its diphosphate groups bridged by Mg^2+^ ions to the glutamates of the Nudix motif. A 1″,2′-glycosidic linkage is observed between the first and second ADPr such that the latter subunit protrudes away from the substrate binding pocket. The second molecule (attached through C1) of ADPr does not make significant contacts to the Nudix fold beyond the mouth of *Hs*NudT16 (*cf*. Fig. 1 and Supplementary Fig. S1). Similar to other structures of di-ADPr bound to enzymes, such as poly(ADP-ribose) glycohydrolase (PDB ID: 4L2H and 5A7R), only the ADP portion of the C1′ linked-ADPr is observed and the non-adenosine ribose at the 1″ aldehyde terminus is disordered with no electron density observed^28,44,66^.

## Discussion

In this study, we sought to understand how *Hs*NudT16 binds and hydrolyzes different forms of ADPr using X-ray crystallography. Crystal structures of *Hs*NudT16 in complex with ADPr further guided our mutagenesis of the enzyme to improve its ability to process PARylated proteins to phosphoribosylated proteins. Since the structure of *Hs*NudT16-ADPr complex demonstrated a key role of the Nudix motif in binding ADPr and in hydrolyzing the phosphodiester bond, we did not mutagenize the Nudix canonical sequence. Rather, we identified key amino acid residues close to binding sites of ADPr and di-ADPr. We made *Hs*NudT16 mutants in hopes of improving the binding to the ADPr moiety and/or by enlarging the substrate-binding groove to allow bulkier substrates, such as protein-conjugated ADPr, to come into closer proximity to the active site. Crystallization was successful for the H24W, F36A, and F61S mutants and the structures helped rationalized the effects of our single-point mutations. For example, the distance between residues Phe36′ and Phe61 increased as a result of the mutations, thus widening the substrate-binding site of *Hs*NudT16. Despite co-crystallization efforts with ADPr, electron density for the substrate could not be seen in the binding site of these mutants. This outcome could be explained by our Michaelis-Menten experiments with ADPr, in which these mutations led to at least a 2-fold increase in *K*_m_ compared to the original *Hs*NudT16.

Demodification assays of PARylated PARP1 showed improved demodification for the *Hs*NudT16 Δ17, F36A, and F61S mutants while the F36A F61S mutant had no significant difference and H24W mutant was catalytically inactive. Although NudT16 processes MARylated substrates *in vitro* (Fig. 3 and ref.^32^), the hydrolysis reaction is not as efficient as PARylated proteins. Under the tested conditions, demodification assays of MARylated PARP10^CD^ showed no clear changes between wild-type *Hs*NudT16 and all mutants except H24W, which had minimal hydrolysis activity. The insignificant demodification activity with H24W mutant with protein-conjugated ADPr as well as its Michaelis-Menten results on hydrolysis of free ADPr could be explained by the position of the tryptophan in the structure, which suggests that H24W occludes the recognition site of the purine. While NudT16 exists as a dimer in solution, functionally active mutant can exist as dimer (*e*.*g*., F61A) or monomer (*e*.*g*., F36A and F36A F61S)

**Figure 5:**
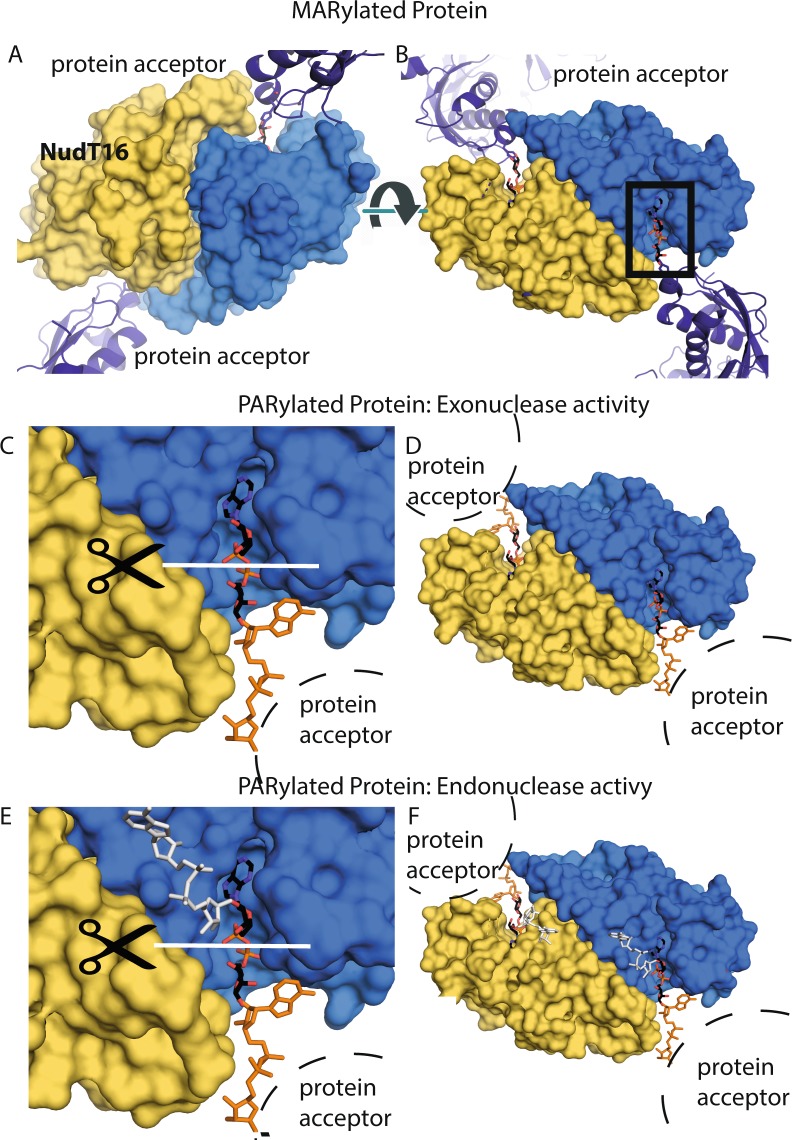
Model of *Hs*NudT16 binds to an ADP-ribosylated protein. (**A**) Surface representation of the *Hs*NudT16 with ADPr bound in the active site attached at the 1″ position to a model substrate at each binding site. Eukaryotic elongation factor 2 was used as a model as it is the only structure of ADP-ribosylated substrate available in the PDB for modeling the interaction. As indicated, protein-conjugated ADPr (MARylated protein) can reach the catalytic site of *Hs*NudT16 without steric hindrance. (**B**) 180° view from (**A)** displaying how an MARylated protein can reach the active site. (**C**) Zoom-in view of the binding site of *Hs*NudT16 shown with an ADPr (black sticks colored according to atom type) to be hydrolyzed and a second ADPr molecule attached at the 1″ position modeled (orange). An arc shows the location of a probable protein acceptor. (**D**) Zoom out view of (**C**). (**E**) Zoom-in view of the binding site of *Hs*NudT16 shown with an ADPr (black sticks colored according to atom type) to be hydrolyzed and a second ADPr molecule attached at the 1″ position modeled (orange), with a third ADPr attached at the 2′-OH position modeled (white). An arc shows the location of a probable protein acceptor. (**F**) Zoom-out view of the binding site of (**E**).

The difference in *Hs*NudT16 demodification efficacy observed between MARylated and PARylated proteins could be a function of steric hindrance (Fig. 5A,B). *Hs*NudT16-di-ADPr structure suggests that the 2′-OH terminus of a PAR molecule is bound by the Nudix motif while the 1″-terminus, which is conjugated to proteins, is distal to the active site (*cf*. Fig. 4E,F). Here we modeled eukaryotic elongation factor 2 as a MARylated substrate (PDB ID: 1ZM2; Fig. 5A,B). As indicated, MARylated protein can reach the catalytic site of *Hs*NudT16 without steric hindrance. Having said that, PARylated proteins would have even easier access to the *Hs*NudT16 catalytic center with less steric hindrance than MARylated proteins where the latter would be closer to residues, such as Phe36′ and Phe61, at the mouth. The width of the mouth of *Hs*NudT16 (10–12 Å) displays the maximum width of the protein feature nearby the ADP-ribosylated sites that can approach the site for hydrolysis. In addition, the orientation of the PAR suggests a potential exonuclease activity from the 2′-OH terminus in concordance with the purported snoRNA decapping activity of *Hs*NudT16^60^ (Fig. 5C,D). However, since there is sufficient space to accommodate an additional ADPr linked to the 2′-OH terminus, we cannot exclude the possibility of endonuclease activity (Fig. 5E,F).

In summary, our structural analyses allowed us to engineer mutants to improve the catalytic efficiency of hydrolyzing PARylated proteins while maintaining the same activity for MARylated proteins as compared to the *Hs*NudT16. This improved capability of mutants may potentially be useful for processing PARylated residues to phosphoribose tag for site identification by mass spectrometry. Given that these mutants have less activity towards free ADPr, such differential activities may also possibly be exploited to investigate the function of free vs. protein-conjugated ADPr.

## Supplementary information

Supplementary Information

## Data Availability

All data generated or analysed during this study are included in this published article (and its Supplementary Information files). Structural data are deposited in Protein Data Bank.
