# Supplementary material for: Structural analyses of NudT16–ADP-ribose complexes direct rational design of mutants with improved processing of poly(ADP-ribosyl)ated proteins
Source: Sci Rep. 2019 Apr 11;9:5940. doi: 10.1038/s41598-019-39491-w (PMC6459841; doi:10.1038/s41598-019-39491-w)
Supplement: Supplementary file 1 — Supplementary Information [file 41598_2019_39491_MOESM1_ESM.pdf]

Supplementary Information

Structural analyses of NudT16–ADP-ribose complexes direct rational design of mutants with improved processing of poly(ADP-ribosyl)ated proteins

Puchong Thirawatananond[1], Robert Lyle McPherson[2], Jasmine Malhi[1], Sara Nathan[1,6], Michael J. Lambrecht[3], Matthew Brichacek[3], Paul J. Hergenrother[3], Anthony K. L. Leung[2,4,5,*], Sandra B. Gabelli[1,4,6,*]

[1]Department of Biophysics and Biophysical Chemistry, Johns Hopkins University School of Medicine, Baltimore, MD 21205.
[2]Department of Biochemistry and Molecular Biology, Bloomberg School of Public Health, Johns Hopkins University, Baltimore, MD 21205, USA.
[3]Department of Chemistry, University of Illinois, Urbana, IL 61801, USA
[4]Department of Oncology, Johns Hopkins University School of Medicine, Baltimore, MD 21287
[5]Department of Molecular Biology and Genetics, Johns Hopkins University School of Medicine, Baltimore, MD 21287
[6]Department of Medicine, Johns Hopkins University School of Medicine, Baltimore, MD 21205

[*]senior and corresponding authors

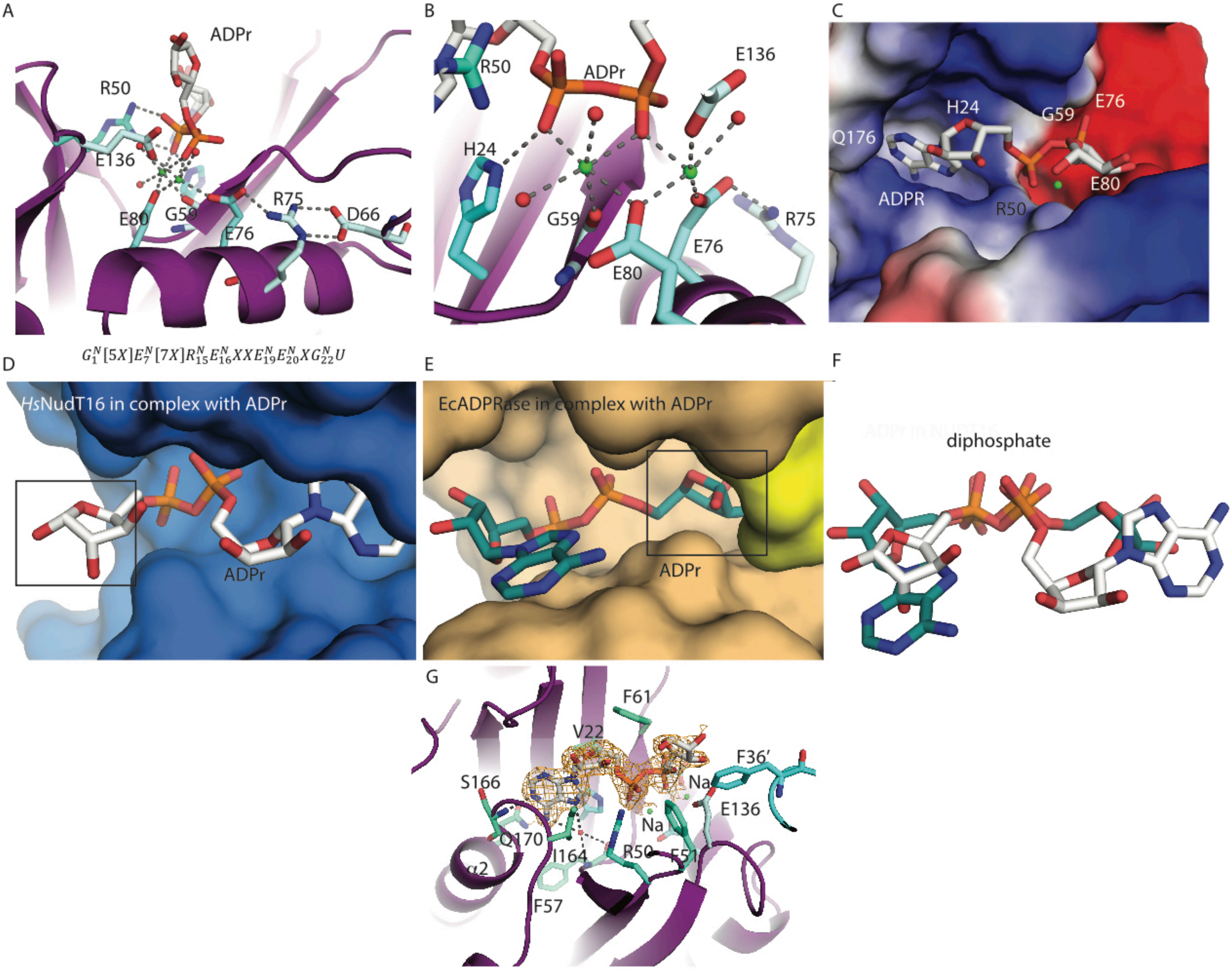

Supplementary Fig. S1. Comparison of *Hs*NudT16 with a member of Nudix ADPrase family. A. Hydrogen bond interactions of the residues of the Nudix signature sequence (light blue) with the secondary structure shown in purple in *Hs*NudT16. is not shown; the conserved glutamic acid in the loop before the helix, is an asparatic acid, D66; are R75 and E76; are E79 and E80. The residue that completes the Mg^{2+} coordination is E136. **B.** Zoom-in view of the magnesium ions (green) that bridge the Nudix signature sequence to the substrate ADPr. **C.** Electrostatic surface map displaying the *Hs*NudT16 charged surface in the ADPr binding site: hydrophobic (colored white) for the adenosine, polar positive (blue) for the ribose, negatively charged (red) for the binding site of the cations that bridge the disphosphates to the protein. The non-adenine ribose is exposed to the solvent. **D.** Surface of the *Hs*NudT16 (marine blue) with the bound ADPr (PDB ID: 5W6X). The box highlights that the non-adenine ribose is exposed and accessible. **E.** Surface of the *Ec*ADPrase (light orange and yellow) with the bound ADPr (PDB ID: 1GQ9). The box highlights that the non-adenine ribose is buried and not accessible to bound ADPr if conjugated to proteins. **F.** Structural overlap of the ADPr as bound in *Ec*ADPRase (white carbon atoms) with the one bound in *Hs*NudT16 (teal carbon atoms) displaying that ADPr is in reverse orientation. The box highlights the overlap in space of the diphosphate. **G.** Omit density map of the ADPr contoured at 1σ (orange) in the same orientation that the 2FoFc of Figure 1B. The secondary structure of *Hs*NudT16 is shown in purple and residues that delimit the binding site are shown as sticks in aquamarine. F36′ from the other monomer is shown in cyan.

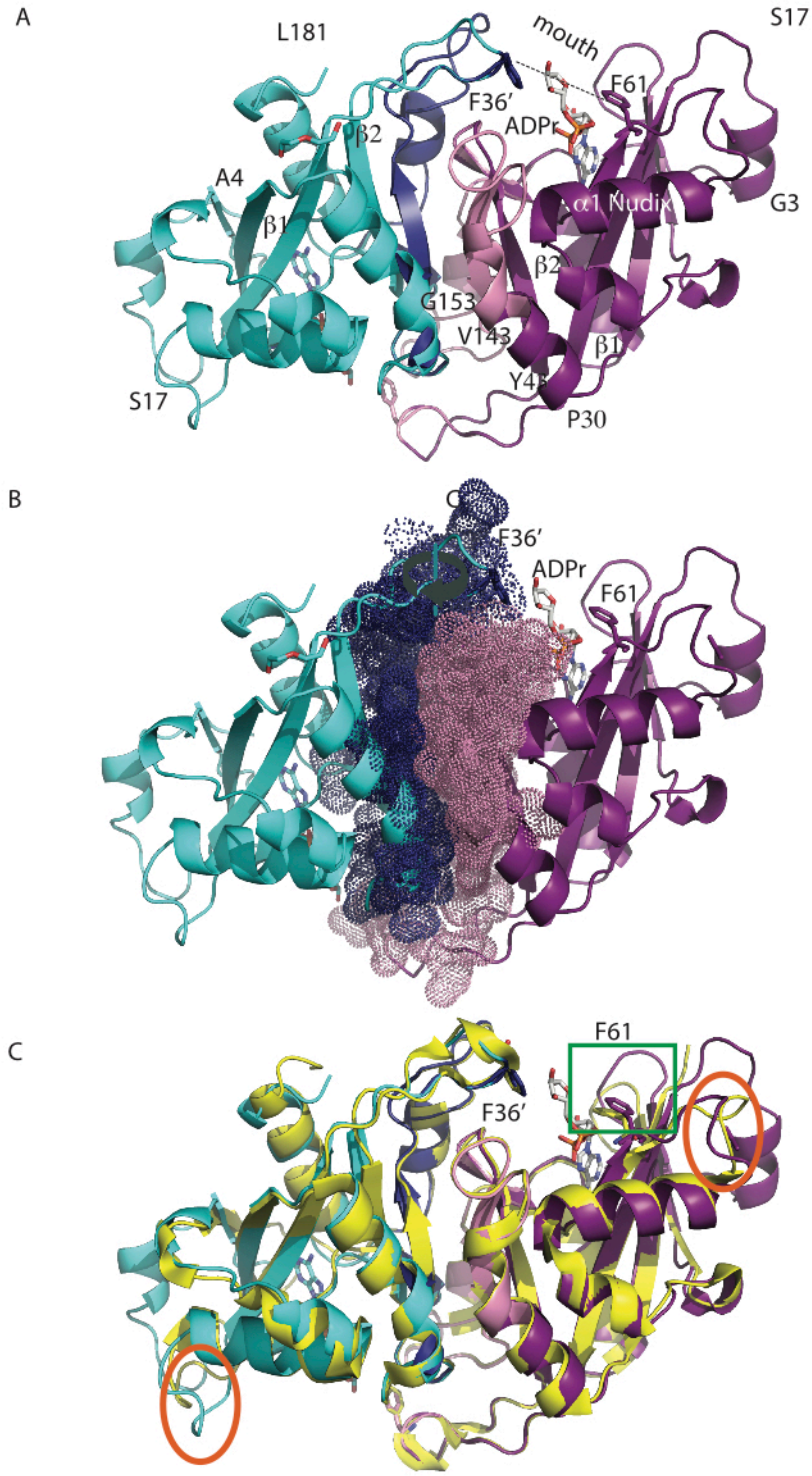

Supplementary Fig. S2. Analysis of the dimer interface of *Hs*NudT16. A. Structure of ADPr bound to the active site of *Hs*NudT16 with one monomer colored in cyan with amino acids involved in the dimer interface in dark blue; the other monomer is in purple with amino acids at the dimer interface in light pink. Residue Phe36′ to Phe 61 of the opposite monomer delineates the "mouth" of the active site. **B.** As in A, but with the residues at the interface in dots. **C.** Structural overlap of the *Hs*NudT16 in complex with ADPr (PDB ID 5W6X) with apo *Hs*NudT16 (PDB ID 3MGM, in yellow). The orange ellipse highlights the differences on amino acids 60-69, (~5 Å) and the green box the different conformation of amino acids 100-110. In the absence of ADPr, Phe61 (yellow) moves in towards the active site where ADPr would bind.

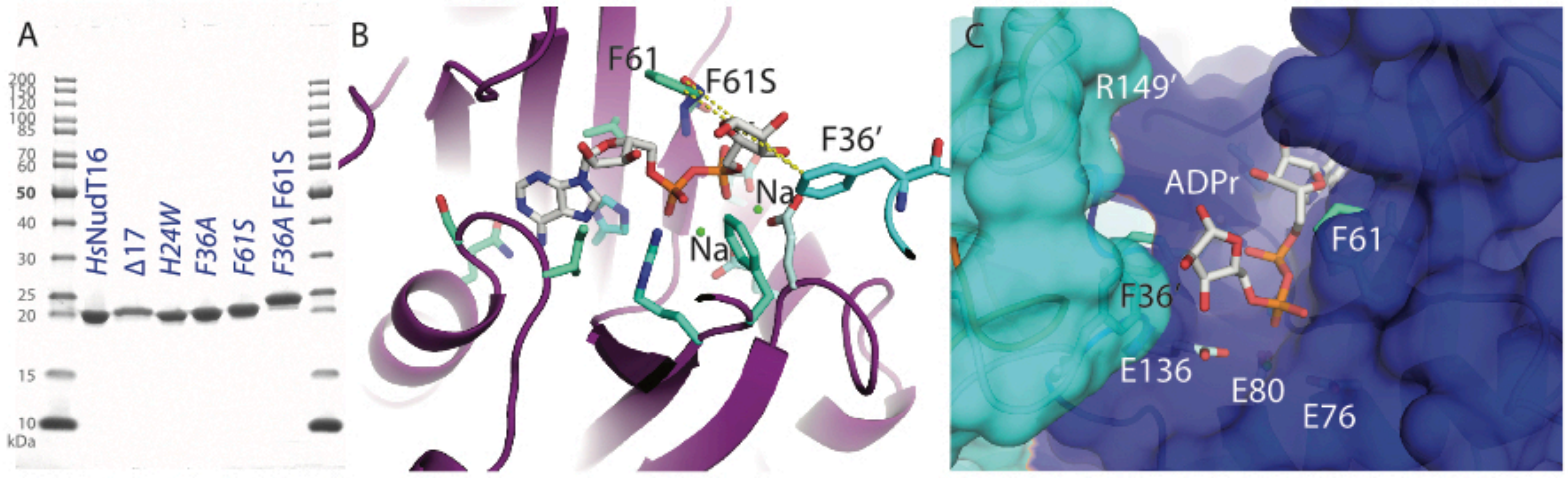

Supplementary Fig. S3. *Hs*NudT16 F61S mutant, like WT, is a dimer, but does not open the binding site significantly. **A.** Coomassie blue-stained gel of *Hs*NudT16 and its mutants. **B.** Amino acids that delimit the ADPr binding site, showed that the F61S distance to F36′ is only one Å longer than the original distance F36′-F61 at the location of the non-adenine ribose. **C.** Surface representation of *Hs*NudT16 F61S (one monomer in marine blue, and another in turquoise blue) in the ADPr binding site showing how the *Hs*NudT16 residue F61 (cyan) protrudes from the mutant and makes the site more open. The ADPr is shown as sticks with white carbon atoms.

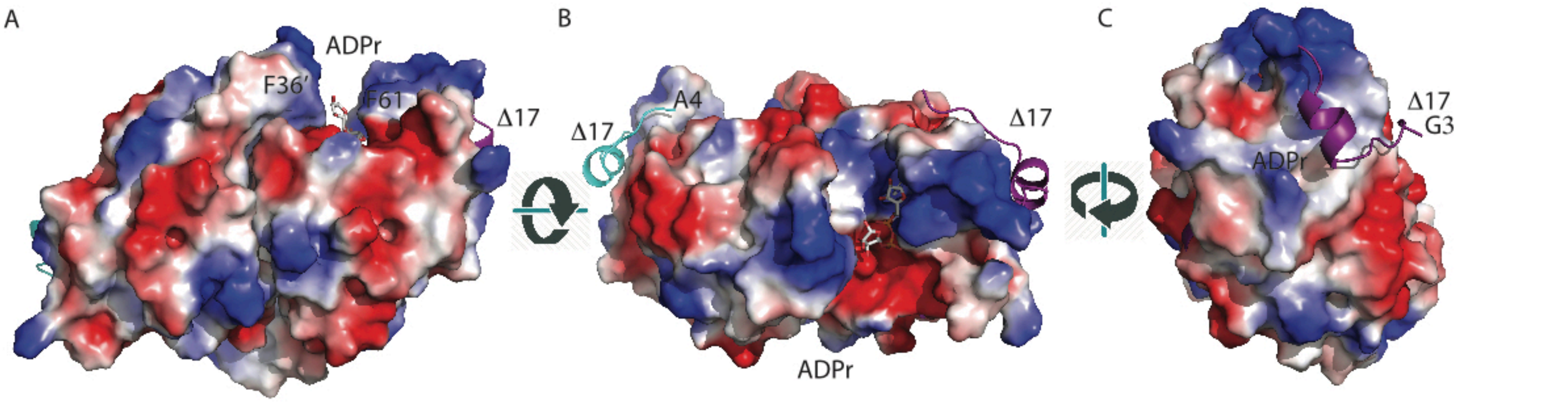

Supplementary Fig. S4. *Hs*NudT16 Δ17 exposes a hydrophobic path on the surface. **A**. Surface representation of the *Hs*NudT16 Δ17 colored according the calculated electrostatic potential in the orientation of Fig. 1A. Residues 3-17 are shown as cartoon in purple and cyan. **B**. 90° rotation to see from the top. **C.** Sideway rotation to observe the hydrophobic surface below the cartoon of the residues that constitute Δ17.

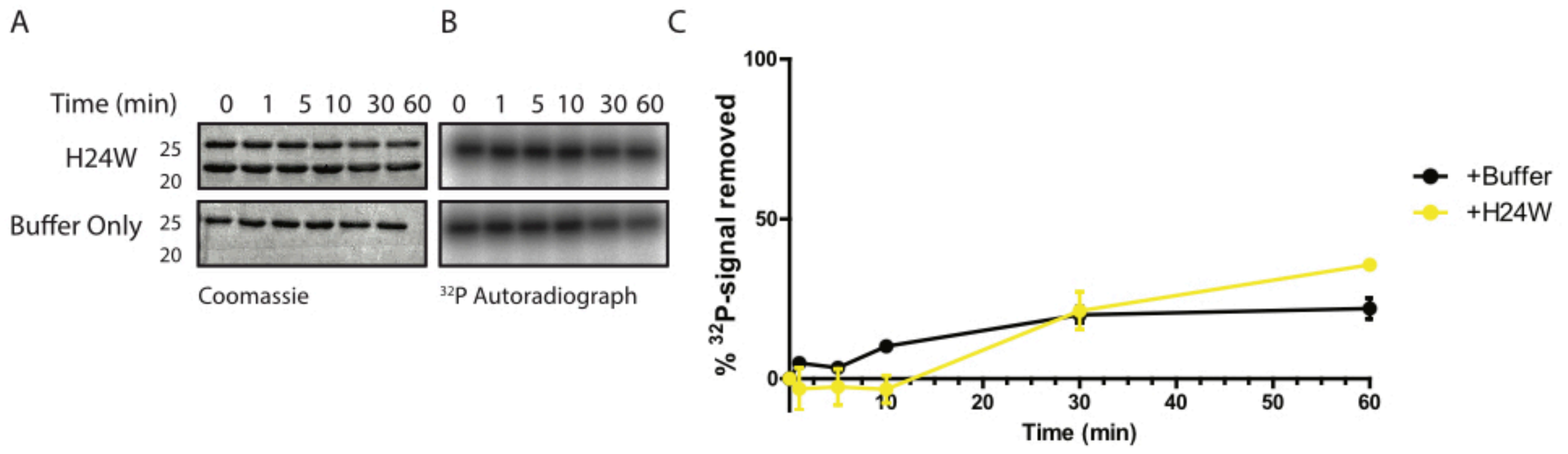

Supplementary Fig. S5. ***Hs*NudT16 H24W mutant displays very little activity towards MARylated proteins. A.** Time course of the demodification reaction of ^{32}P-MARylated PARP10^{CD} by *Hs*NudT16 H24W or buffer alone. **B.** ^{32}P autoradiograph of the same demodification reaction shown in **A.** **C.** Quantification of the removal of ^{32}P-radioactive signal from the time-dependent PARP1 demodification assay by *Hs*NudT16 H24W or buffer alone, n=3.

Figure 3A

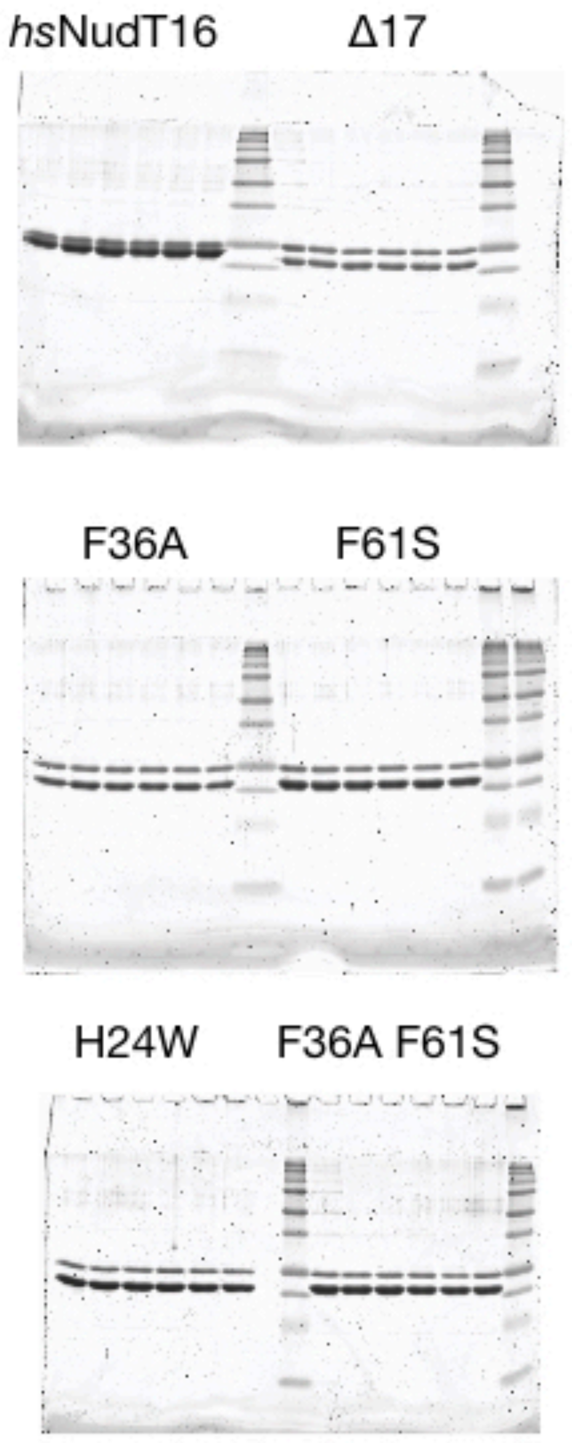

Figure 3B

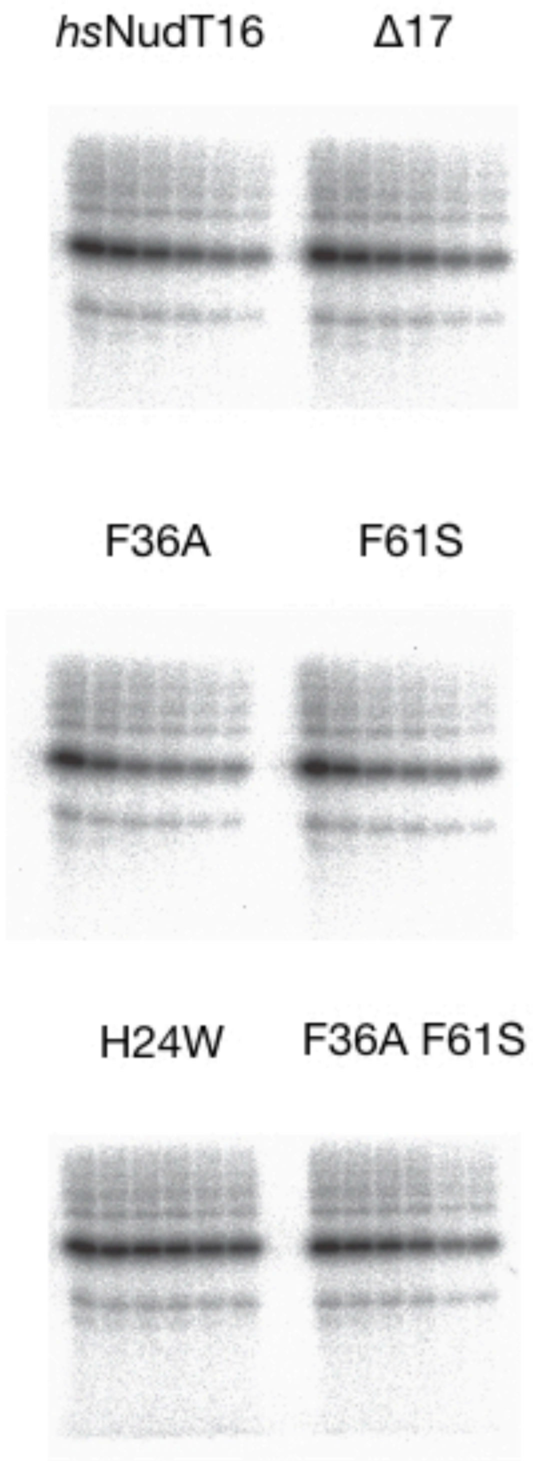

Figure 4A

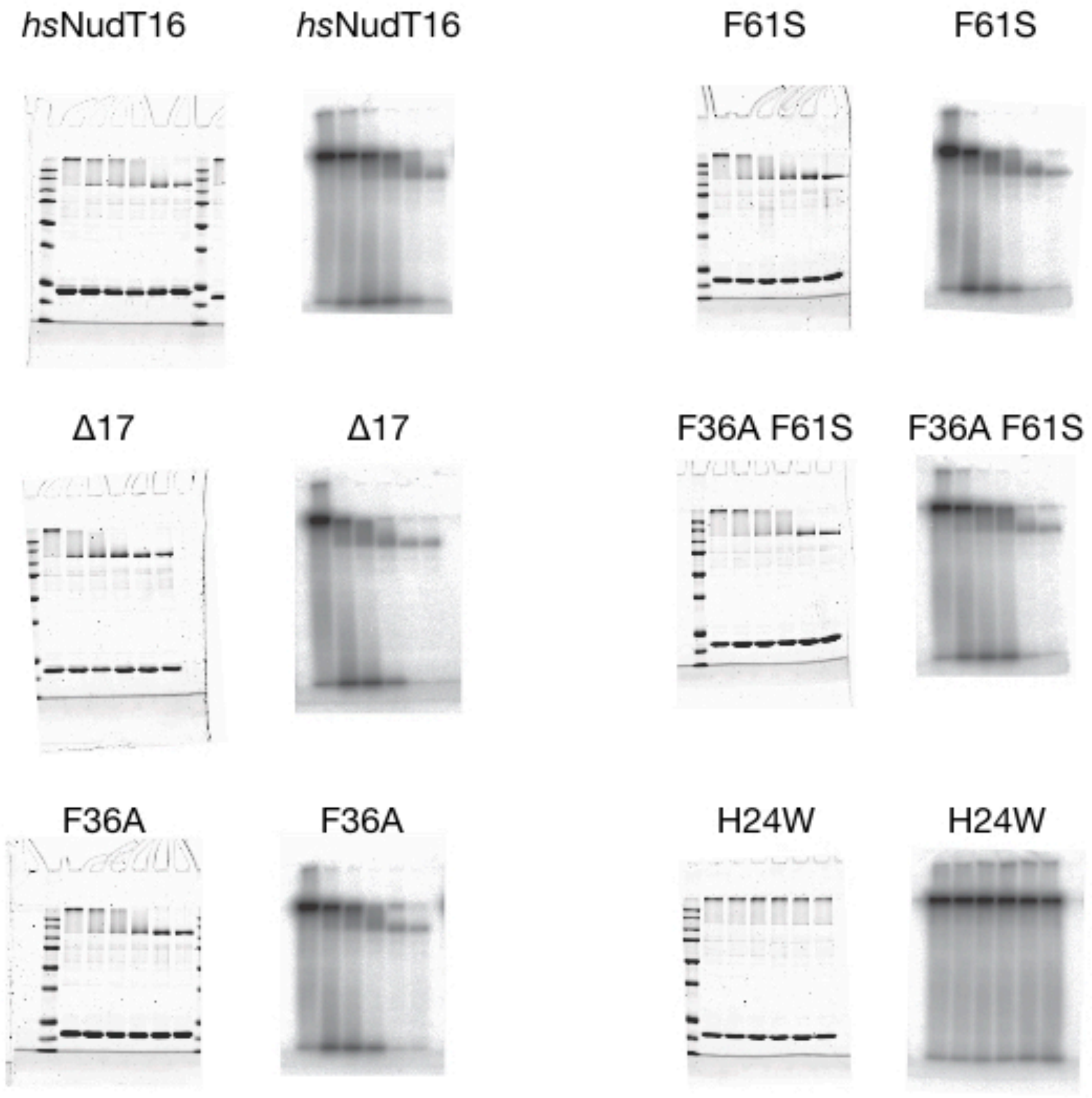

Figure 4C

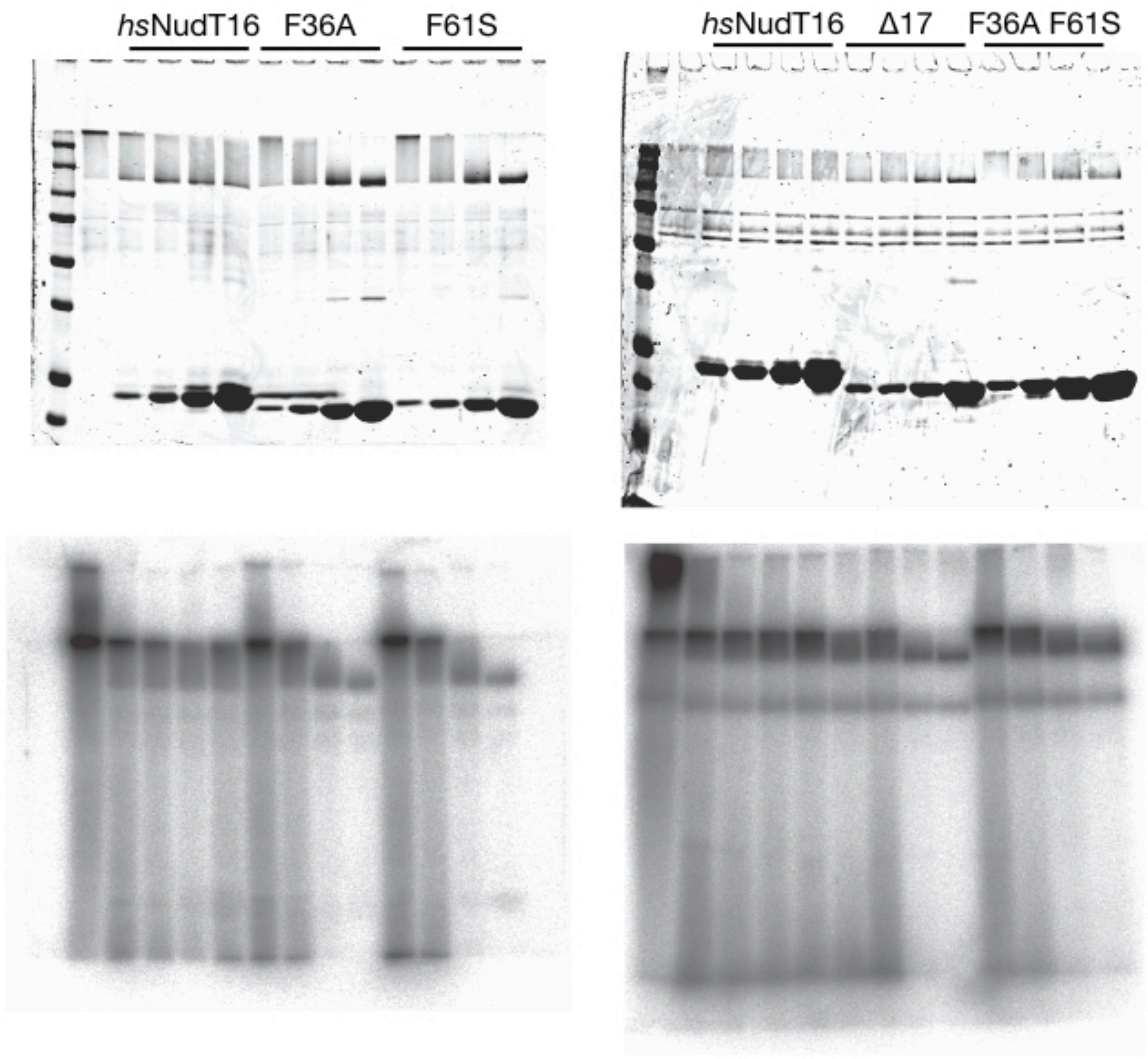

Supplementary Fig. S6. Original gel images for Figures 3 and 4.
